# Biomimetic cellulose-based superabsorbent hydrogels for treating obesity

**DOI:** 10.1038/s41598-021-00884-5

**Published:** 2021-11-01

**Authors:** Marta Madaghiele, Christian Demitri, Ivo Surano, Alessandra Silvestri, Milena Vitale, Eliana Panteca, Yishai Zohar, Maria Rescigno, Alessandro Sannino

**Affiliations:** 1 Department of Engineering for Innovation, University of Salento, Via per Monteroni, 73100 Lecce, Italy; 2 IRCCS Humanitas Research Hospital, Via Manzoni 56, 20089 Rozzano Milan, Italy; 3 Gelesis, Boston, MA 02116 USA; 4 Gelesis, Calimera, Lecce, 73021 Italy; 5 Department of Biomedical Sciences, Humanitas University, Via Rita Levi Montalcini 4, 20072 Pieve Emanuele, Milan, Italy

**Keywords:** Bioinspired materials, Obesity, Bioinspired materials

## Abstract

In the treatment of obesity, nutritional and behavioral modifications are difficult to implement and maintain. Since vegetable consumption is a fundamental part of many dietary interventions and daily nutrient requirements, we developed a novel cellulose-based superabsorbent hydrogel (CB-SAH) platform, inspired by the composition and mechanical properties of raw vegetables, as a mechanobiological therapy. The CB-SAHs properties were studied in a simulated gastrointestinal environment, while their impact on gut tissue was investigated by an ex vivo organ culture (EVOC) model. Functional fibers and raw vegetables were used as reference. CB-SAHs demonstrated orders of magnitude higher elasticity in comparison to the tested functional fibers, however performed similar to the tested raw vegetables. Notably, the biomimetic CB-SAHs with elasticity levels similar to raw vegetables showed benefits in preserving and regulating the gut tissue in the EVOC model. Non-systemic oral mechanotherapeutics based on this technology were advanced through clinical studies, with a first product cleared as an aid for weight management in the US and Europe.

## Introduction

Obesity is recognized as a worldwide epidemic, affecting both adults and children^1,2^. According to recent estimates by the World Health Organization (WHO), the prevalence of obesity has nearly tripled in the last 40 years^1^. Obesity is a major risk factor for a range of diseases, such as cardiovascular diseases, type 2 diabetes, hypertension and several cancers, and it is often linked to depression and social discrimination^3,4^. Although there are likely to be multiple concurrent causes for the rise in obesity, diet is believed to play a primary role^5–7^ due to both the increased availability of highly processed, energy-dense and palatable foods and beverages^5^ as well as the inadequate intake of dietary fibers (DFs) in the Western diet^8,9^. DFs are defined as non-starch polysaccharides (e.g. cellulose) that constitute most of the dry matter of plant foods, such as vegetables, fruits and whole grains^10–12^. Speculations on the evolution of the human diet suggest that the human gastrointestinal (GI) system is adapted to high fiber consumption^8^, and the shift from diets rich in vegetables and fruits to diets rich in animal proteins and refined grains is at least partly responsible for the obesity epidemic^8,9^.

The increased consumption of vegetables and fruits is widely acknowledged as a useful dietary intervention for excess weight^13,14^. While being a rich source of vitamins, minerals and phytonutrients^11,12^, vegetables and fruits also facilitate the adherence to a reduced calorie dietary regimen mostly due to their high DFs and water content^13,14^. They occupy volume in the stomach and contribute to reducing the caloric density or energy density of a meal (i.e. the amount of calories per gram of food)^15^. When transitioning through the upper GI tract, they increase the elasticity (i.e. solid-like firmness) of the GI content, as DFs are not degraded or absorbed^10^. These features are known to enhance satiety^10,15,16^, thereby decreasing the total amount of food intake. They also slow down the absorption of nutrients, thus potentially improving glycemic control^10,17,18^. Further satiety-inducing and health-promoting effects occur once they reach the large intestine, where DFs can be fermented to various extents by the gut microbiota, with the production of short chain fatty acids (SCFAs)^10,19^. SCFAs are valuable compounds that are used by colonic cells as an energy source and participate in the modulation of gut homeostasis and gut hormones known to regulate the appetite^10,19,20^. Notably, mounting evidence also suggests that the physical form of vegetables and fruits affects their satiating action, with solid forms (especially in the raw state) being more effective than liquid ones^21,22^. This finding has been attributed in part to the extent and duration of gastric distension and GI transit^7,10,23^.

**Figure 1:**
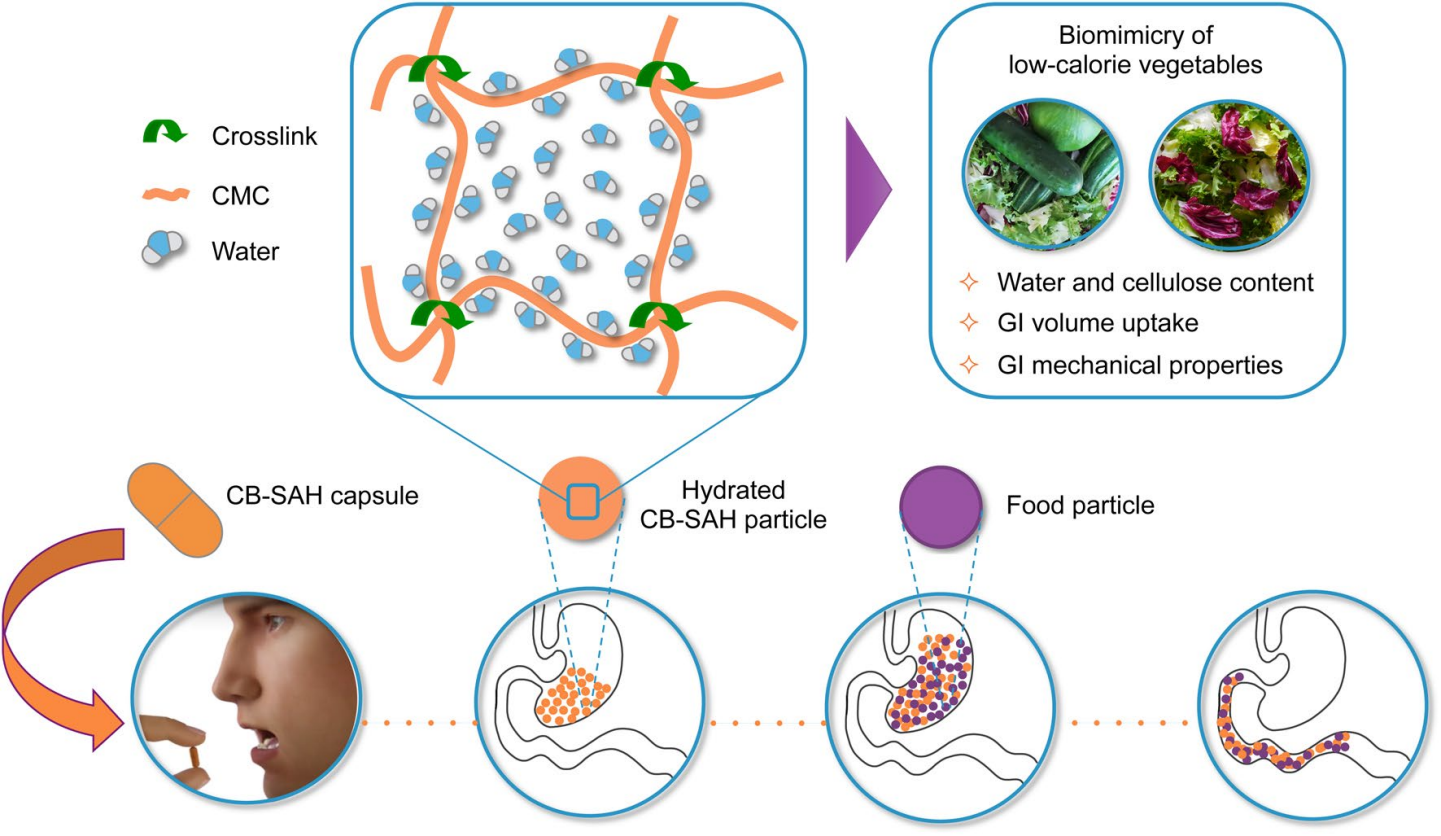
Design of biomimetic CB-SAHs for weight management. Taking inspiration from basic compositional and structural properties of raw vegetables while in transit through the GI tract, we designed novel CB-SAHs based on crosslinked carboxymethylcellulose sodium salt (CMC). The CB-SAHs are orally administered in capsules, hydrate in the stomach, mix up with the ingested food, and move along the small intestine without being absorbed. Once in the colon (not depicted in the scheme), they undergo partial degradation to return the absorbed water to the body, while the cellulosic material is excreted with the feces.

While diet and exercise represent the first steps to take for treating obesity and excess weight, nutritional and behavioral modifications, such as increasing the consumption of raw vegetables, are often difficult to implement and maintain^24,25^. Therefore, our aim was to design an orally administered hydrogel for weight management, inspired by basic compositional and structural features of raw vegetables (Fig. 1). Several known volumetric approaches focus on the stomach (e.g. gastric balloons^26^). Instead, our goal was to increase the volume and elasticity of the ingested foods throughout the entire digestive system, without additional caloric value. Such a non-absorbed hydrogel could both reduce the caloric density of the meals and also increase the elasticity of the ingested foods in a similar manner to raw vegetables. We hypothesized that such a biomimetic approach could have a favorable efficacy and safety profile. A high absorbance capacity, distinctive of superabsorbent hydrogels, is a key requirement for both ease of use and practicality reasons. First, we needed a practical number of capsules to establish an effective orally administered dose. Only a superabsorbent material could allow for this. Second, to mimic the partial degradation of vegetables in the colon, we sought to develop a hydrogel susceptible to a similar degradation process, promoted by colonic bacterial enzymes, before being eliminated in the feces. This partial degradation is important, as it allows the release of most of the carried water, which is then re-absorbed in the colon, thus reducing the potential risk of dehydration or diarrhea.

To this purpose, we have developed a technological platform of cellulose-based superabsorbent hydrogels (CB-SAHs), to the best of our knowledge the first reported superabsorbent technology that utilizes only Generally Recognized as Safe (GRAS) building blocks used in foods. These CB-SAHs are exclusively based on carboxymethylcellulose sodium salt (CMC) as the polymer backbone (used as a thickening agent in foods^27^), and citric acid (CA) as the crosslinking agent (found in citrus fruits). Previous studies on the synthesis of cellulose-based hydrogels^28,29^ concluded that combining non-polyelectrolyte (e.g. hydroxyethylcellulose, HEC) to polyelectrolyte backbones (e.g. CMC) is necessary to achieve intermolecular rather than intramolecular crosslinks, which are fundamental to create stable superabsorbent networks^30,31^. Here, however, we present a tunable CB-SAH platform that is based on CMC as the only polymer backbone.

In order to identify the best hydrogel candidates for further clinical development, we synthesized and studied CB-SAHs with different levels of elasticity. The mechanical properties of the CB-SAHs were assessed in simulated GI conditions, and their potential effect on the gut tissue health was evaluated utilizing an ex vivo organ culture model. In both of these studies several vegetables and functional fibers were used as a reference.

## Results

### Preparation of CB-SAHs with tunable elasticity from CMC backbones

**Figure 2:**
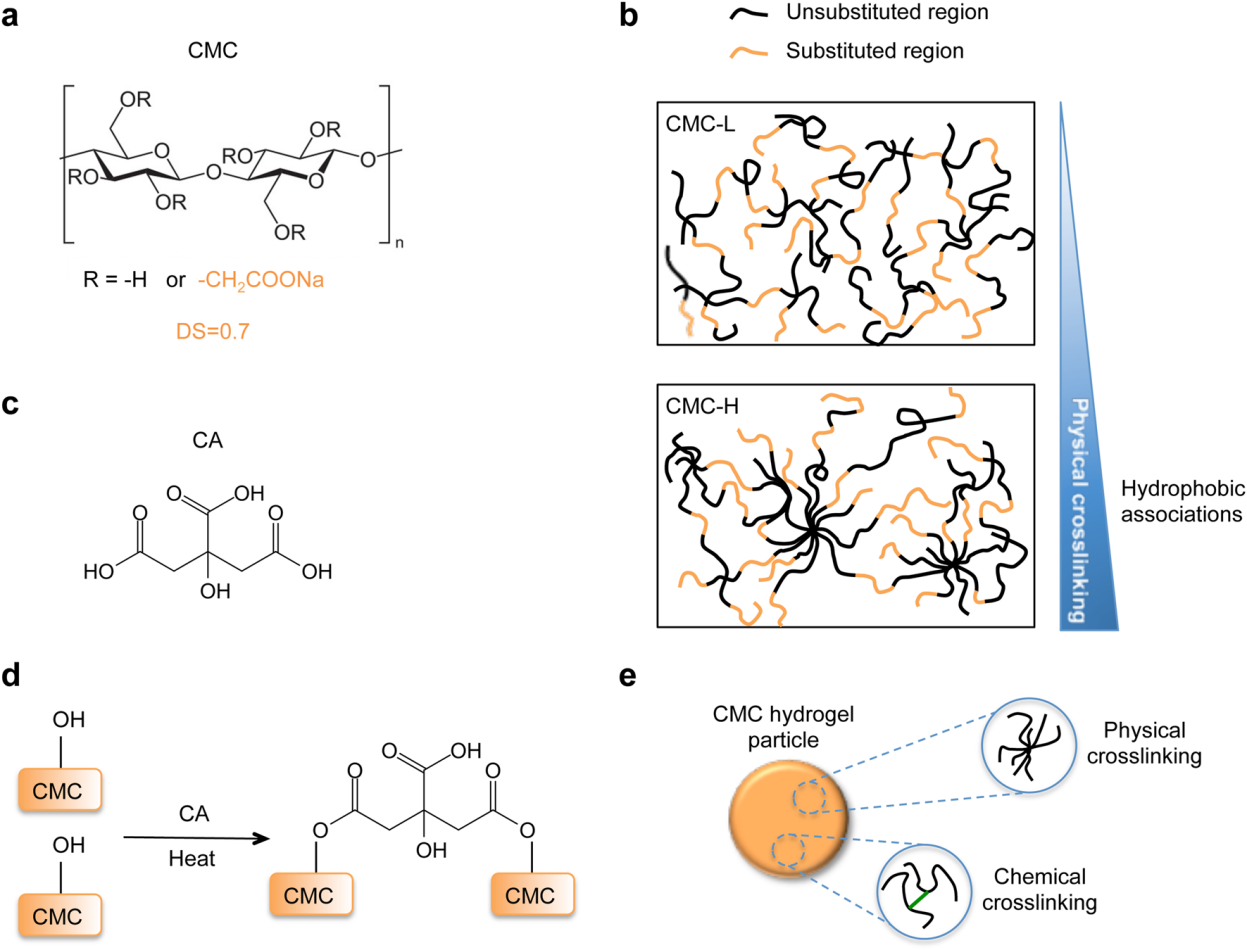
Preparation of stable CMC networks by the combination of physical and chemical crosslinking. (**a**) Chemical structure of the CMC repetitive unit; two CMCs with DS = 0.7 were used in this work, i.e. CMC-L and CMC-H. (**b**) CMCs with DS ≤ 1 may form physical crosslinks in concentrated solutions, due to the establishment of hydrophobic associations among the unsubstituted cellulose blocks; based on its higher molecular weight and higher thixotropy, CMC-H is expected to form a more entangled structure than CMC-L (at fixed concentration). (**c**) Chemical structure of CA and (**d**) esterification reaction with the CMC; the reaction is activated by heat and occurs upon preliminary casting and drying of a CA/CMC solution. (**e**) Presence of both physical and chemical crosslinks in the final CMC network.

Among cellulose derivatives, CMC is an optimal candidate for the synthesis of CB-SAHs that are expected to function in the wide range of pH and ionic strength conditions found along the GI tract. CMC is modified cellulose containing a given number of carboxymethyl groups per glucose unit, as defined by the degree of substitution (DS) (Fig. 2a). As well known, carboxymethyl groups dissociate in aqueous solution, thus providing the CMC chains with anchored negative electrostatic charges. These fixed ions make a CMC-based crosslinked material not only superabsorbent, due to the Donnan effect^30^, but also responsive to pH and ionic strength variations, as these environmental variables control the degree of ionization of the CMC chains and the number of mobile counterions in the bathing solution^30^. However, the chemical crosslinking or stabilization of single CMC in water solution is hindered by the electrostatic repulsion among the CMC chains, which leads to the formation of intramolecular rather than intermolecular crosslinks^28^. Intramolecular crosslinking gives rise to weak polymer networks with poor structural integrity and low elasticity.

However, in case of DS ≤ 1, physical crosslinking of concentrated CMC solutions may occur under static conditions (i.e. at rest), due to the establishment of hydrophobic associations among the unsubstituted cellulose blocks (Fig. 2b)^32^. Such physical associations or entanglements, which are responsible for the observed thixotropy of CMC solutions (i.e. time-dependent flow behavior, characterized by shear thinning and structural recovery after given time at rest)^27,32^, are favored when the CMC has a higher molecular weight and/or is less uniformly substituted (‘blocky’ CMCs exhibit a higher tendency to form hydrophobic aggregates than regularly substituted ones)^32,33^.

In the search of a method that could promote the formation of stable CMC networks, without the use of any additional ‘reinforcing’ agents (e.g. non-polyelectrolytes^28,29^ or cyclodextrins^34^), we focused on the optimization of CA-based crosslinking^35^ (Fig. 2c, d). Since the crosslinking reaction (i.e. an esterification between the carboxylic groups of CA and the hydroxyl groups of cellulose) occurs upon preliminary casting and drying of a CA/CMC solution^35^, we hypothesized that a stable CMC network could form as a result of both chemical and physical crosslinks, with the latter adding more elastically effective junction points to the covalent network (Fig. 2e). In this approach, physical crosslinks are controlled by the use of given CMC types, e.g. having given molecular weight distribution and/or uniformity of substitution, while chemical crosslinks are modulated by the CA crosslinking.

Here, we used two commercially available CMC types, referred to as CMC-L and CMC-H, to produce different classes of CB-SAHs with variable elasticity, namely GelA and GelB (see Table 1 and Methods). Although both CMCs have a nominal DS of 0.7, we found that they greatly differ for the molecular weight distribution and the thixotropic behavior of their aqueous solutions (see Supplementary Methods and Supplementary Figs. 1, 2). In particular, CMC-H shows a higher molecular weight (M_w_), a lower polydispersity index (PDI) and a larger thixotropy than CMC-L. This suggested the potential of CMC-H chains to form a more entangled structure compared to CMC-L ones (Fig. 2b).

**Table 1:** Synthesis of CB-SAHs with tunable elasticity.

CB-SAH class	CB-SAH code	CMC type	M_W_* (Da)	PDI*	TI* (%)	CA/CMC (% w/w)	Crosslink time @120 °C (h)
GelA	GelA	CMC-L	2.0 × 10^6^	11.1	21.3	0.3	8
GelB	GelB01	CMC-H	2.5 × 10^6^	4.2	29.6	0.2	0
	GelB02						4
	GelB03						6
	GelB04						8

Different CMC types and CA crosslinking parameters were adopted to control the extent of physical and chemical crosslinking in the resulting CB-SAHs.

Details on the CMC characterization are provided in the Supplementary Methods.

*For each CMC type, values of M_w_ and polydispersity (PDI) were evaluated by size exclusion chromatography, while values of thixotropic index (TI), calculated as described previously^27^, were estimated by means of hysteresis loop tests on 2% w/v aqueous CMC solutions.

**Figure 3:**
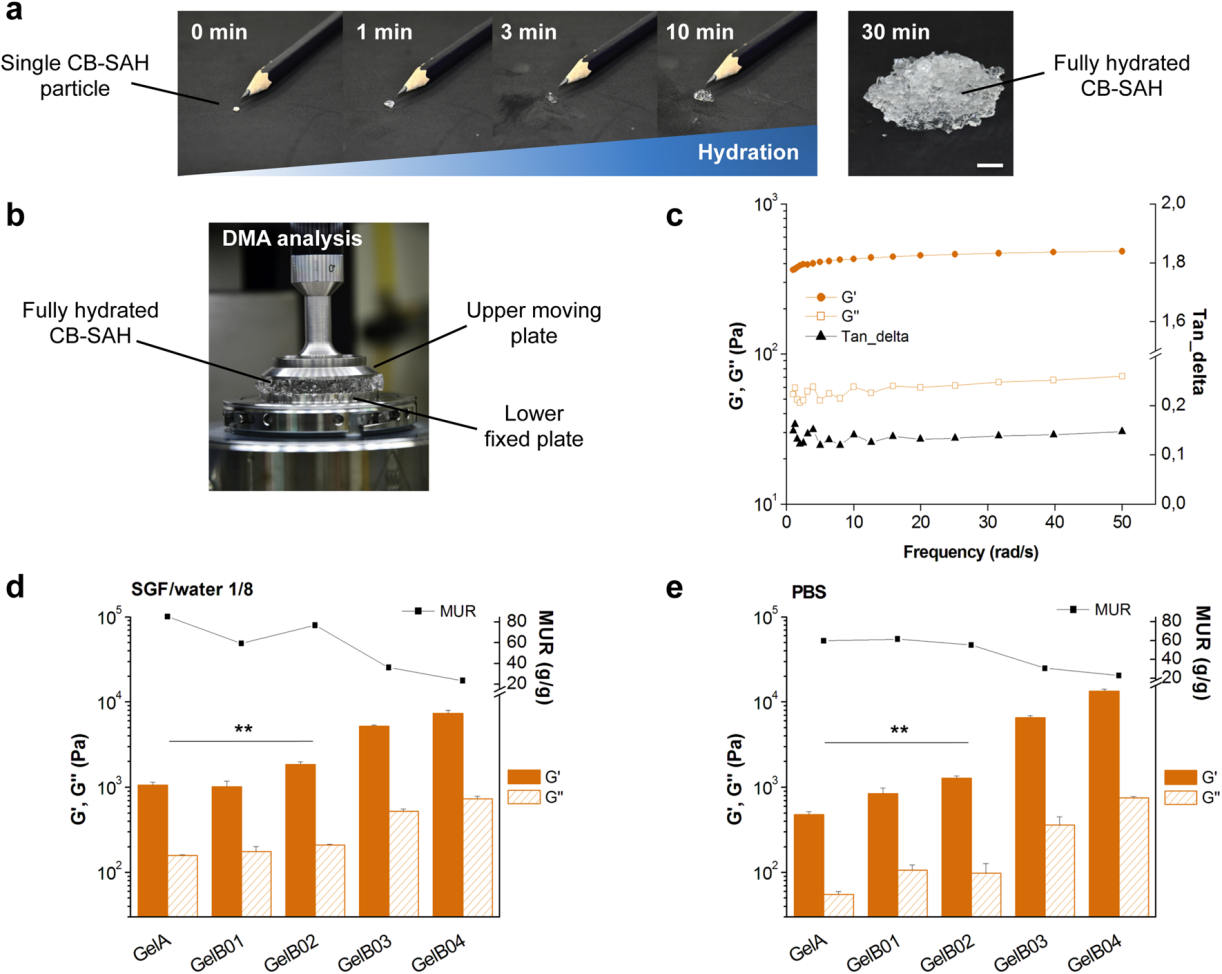
CB-SAHs absorption (MUR) and elasticity (G′) in different aqueous media. (**a**) Hydration kinetics of a single CB-SAH particle (GelA in SGF/water 1/8 v/v), observable as a progressive increase in size with respect to a pencil tip, and visual appearance of the fully hydrated CB-SAH (0.25 g of GelA particles, with a size range 100–1000 µm, in SGF/water 1/8 v/v; scale bar 1 cm). (**b**) Set-up of the DMA analysis to measure the elastic modulus G′ and the viscous modulus G″ of the hydrated CB-SAH. (**c**) Exemplary DMA curves obtained for the CB-SAHs (GelA), indicating G′, G″ and the ratio G″/G′ (tan_delta), as a function of frequency. (**d**) G′ and G″ (at 10 rad/s) and MUR of the CB-SAHs upon incubation in SGF/water 1/8 v/v. (**e**) G′, G″ (at 10 rad/s) and MUR of the CB-SAHs upon incubation in PBS. In (d) and (e), results are the mean ± SD of three independent measurements. Plots in (**c**), (**d**) and (**e**) were produced with Origin 6.0, www.originlab.com. The different G′ of GelA and GelB02 is highlighted by **(*p* < 0.01).

GelA and GelB samples were prepared in granular form, with a particle size range of 100–1000 μm in order to rapidly hydrate and create gel pieces similar in size to ingested raw vegetables (see Methods and Fig. 3a, Supplementary Movie 1). Right after the synthesis, we evaluated the actual formation of CB-SAHs by assessing the dynamic-mechanical properties (Fig. 3b) and the absorption of the granular materials in two different media, i.e. a mixture of simulated gastric fluid (SGF) and water, with SGF/water 1/8 v/v (diluted SGF, pH = 2.1; Supplementary Table 1) and phosphate buffered saline (PBS) (pH = 7.4).

In agreement with the expected formation of crosslinked networks, all hydrated samples showed a prevalently elastic behavior, with the elastic modulus G′ (in the following also referred to as elasticity) being one order of magnitude higher than the viscous modulus G″ over the tested frequency range (Fig. 3c). For the sake of comparison, values of G′ and G″ (at 10 rad/s), together with the corresponding medium uptake ratio (MUR), are reported in Fig. 3d, e for hydration in diluted SGF and PBS, respectively.

Interestingly, GelB02 (based on CA-crosslinked CMC-H, Table 1) showed increased elasticity over GelA (based on CA-crosslinked CMC-L), regardless of the hydration medium. In detail, the G′ values of GelA and GelB02 were respectively 1066 ± 92 Pa and 1846 ± 143 Pa in diluted SGF (Fig. 3d; *p* < 0.01), and 479 ± 44 Pa versus 1292 ± 68 Pa in PBS (Fig. 3e; *p* < 0.01). The significant increase of elasticity for GelB02 is directly ascribable to the use of CMC-H instead of CMC-L, which favors the formation of a more entangled polymer structure. However, the MUR of GelB02, although high, was reduced compared to GelA (GelA vs. GelB02: 85.3 ± 1.1 g/g vs. 77.0 ± 1.0 g/g in diluted SGF, *p* < 0.0001; 59.7 ± 1.1 g/g vs. 55.0 ± 0.6 g/g in PBS, *p* < 0.0001). We also observed that GelA and GelB02 showed increased elasticity at higher MUR levels, i.e. in diluted SGF. The extension of the polymer chains resulting from the medium uptake promoted the elastic response of the crosslinked network. Moreover, the significantly higher MUR values achieved in diluted SGF compared to PBS (*p* = 0.02 for GelA; *p* = 0.03 for GelB02) highlighted the marked sensitivity of GelA and GelB02 to the ionic strength of the bathing solution (13 mM of diluted SGF vs. 162.7 mM of PBS). In this regard, the presence of additional ionizable groups (-COOH) in the CA-crosslinked networks (Fig. 2d) could further enhance the environmental responsiveness of the CMC backbone.

On the contrary, GelB01 (which was not stabilized by CA crosslinking, Table1) showed elasticity and absorption values that were not substantially affected by the hydration medium (Fig. 3d, e). As for GelB03 and GelB04 (obtained by progressively increasing the CA crosslinking time), they showed lower MUR and higher G′ values with respect to GelB02, in both hydration media, in accordance with the expected formation of increasingly crosslinked covalent networks (Fig. 3d, e). In particular, GelB03 and GelB04 showed higher elasticity (as well as lower absorption) in PBS (Fig. 3e), compared to SGF (Fig. 3d). In PBS, the G′ values of GelB03 and GelB04 were respectively 6550 ± 372 Pa and 13,482 ± 653 Pa, while being 5216 ± 147 Pa and 7318 ± 620 Pa in diluted SGF.

Overall, we found that the CB-SAHs elasticity was tunable, particularly by changing the CMC type and/or adjusting the CA crosslinking. Among the tested samples, GelA and GelB02 showed different elasticity levels, coupled with high hydration in diluted SGF (about 70–80 times their dry weight). These samples were thus selected for further characterization and development.

### CB-SAHs absorption and elasticity in simulated GI conditions

An in vitro GI model was specifically designed to simulate the passage of the CB-SAHs, as well as several references (e.g. raw vegetables), through the different parts of the GI tract (i.e. stomach, small intestine and colon). The designed model allowed to assess, in a reproducible system, the expected variations of MUR (absorption) and G′ (elasticity) of the tested materials through the GI tract (see Methods and Supplementary Table 2 for details).

**Figure 4:**
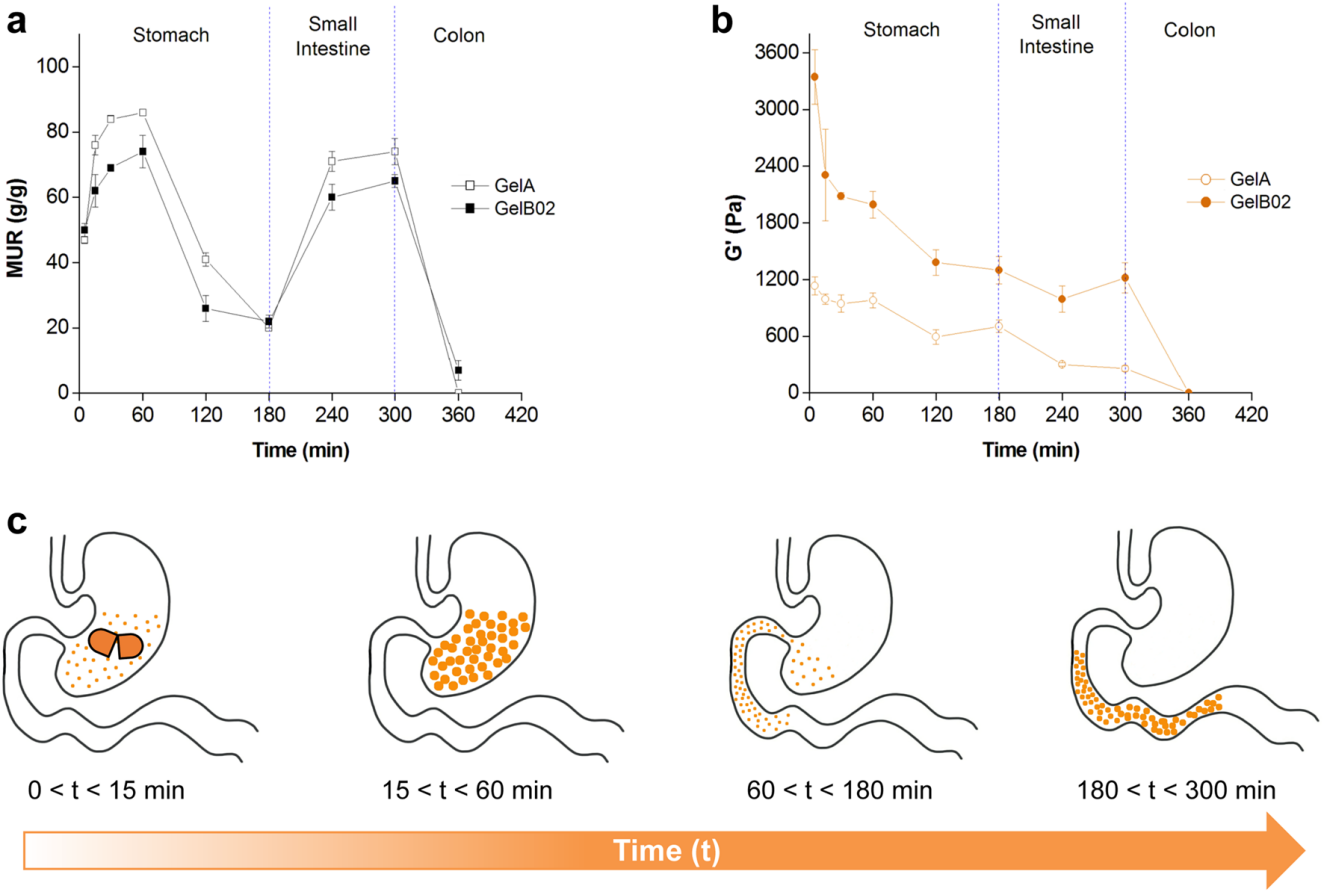
CB-SAHs absorption (MUR) and elasticity (G′) in simulated GI conditions. (**a**) Variations of MUR and (**b**) elastic modulus G′ of GelA and GelB02, as assessed by the in vitro simulated GI model (n = 3 at each time point, mean ± SD; plots produced with Origin 6.0, www.originlab.com). (**c**) Graphical sketch of a CB-SAH hydrating in the stomach and then traveling through the intestine, as simulated by the in vitro model here used.

GelA reached a higher hydration in SGF/water 1/8 v/v (pH 2.1) than GelB02, with MUR values, at 60 min of incubation, of about 86 g/g and 74 g/g for GelA and GelB02, respectively (*p* < 0.0001, Fig. 4a). Following incubation in SGF/water 1/4 v/v (pH 1.8) up to 120 min and then in pure SGF (pH 1.1) for an additional hour, the MUR of both CB-SAHs gradually diminished (down to 20 g/g and 22 g/g for GelA and GelB02, respectively; *p* = 0.47), consistently with the lower hydration expected at decreasing pH values^30^. Then, the further pH increase upon immersion in simulated intestinal fluid (SIF, pH 6.8) led to the re-hydration of both CB-SAHs: at 300 min, the MUR of GelA and GelB02 increased up to the 80–85% of the initial value in SGF/water 1/8 v/v (*p* < 0.0001 for GelA at 300 min vs. GelA at 60 min; *p* < 0.01 for GelB02 at 300 min vs. GelB02 at 60 min). The final incubation of the CB-SAHs in simulated colonic fluid (SCF) then induced a rapid decrease of the MUR, due to concurrent degradation of the CMC network.

In accordance with the hydration kinetics, the G′ modulus of both GelA and GelB02 (Fig. 4b) reached its maximum value upon incubation in SGF/water 1/8 v/v, with GelB02 showing a significantly higher modulus than GelA (980 Pa and 1990 Pa for GelA and GelB02 respectively, at 60 min of incubation; *p* < 0.0001). The pH decrease resulting from the subsequent incubation in SGF/water 1/4 v/v and then in pure SGF, which led to a lower hydration, also caused a gradual decrease of the elasticity for both CB-SAHs. In particular, at 180 min of incubation, a reduction of G′ of about 28% and 35% was detected for GelA and GelB02, respectively (*p* < 0.05 for GelA at 180 min vs. GelA at 60 min; *p* < 0.0001 for GelB02 at 180 min vs. GelB02 at 60 min). A gradual reduction of G′ (down to approximately 260 Pa at 300 min) was then found for GelA upon incubation in SIF. In particular, G′ diminished from 180 to 240 min (*p* < 0.01) and then remained quite stable up to 300 min (*p* = 0.72). On the contrary, GelB02 was quite stable in SIF, with its elastic modulus at 300 min (~ 1200 Pa) being almost the same at 180 min (*p* = 0.58). Finally, consistently with the more extensive backbone degradation expected in the colon, the G′ modulus of both CB-SAHs rapidly diminished in SCF.

### Mechanical properties comparison with raw vegetables and functional fibers in simulated GI conditions

**Figure 5:**
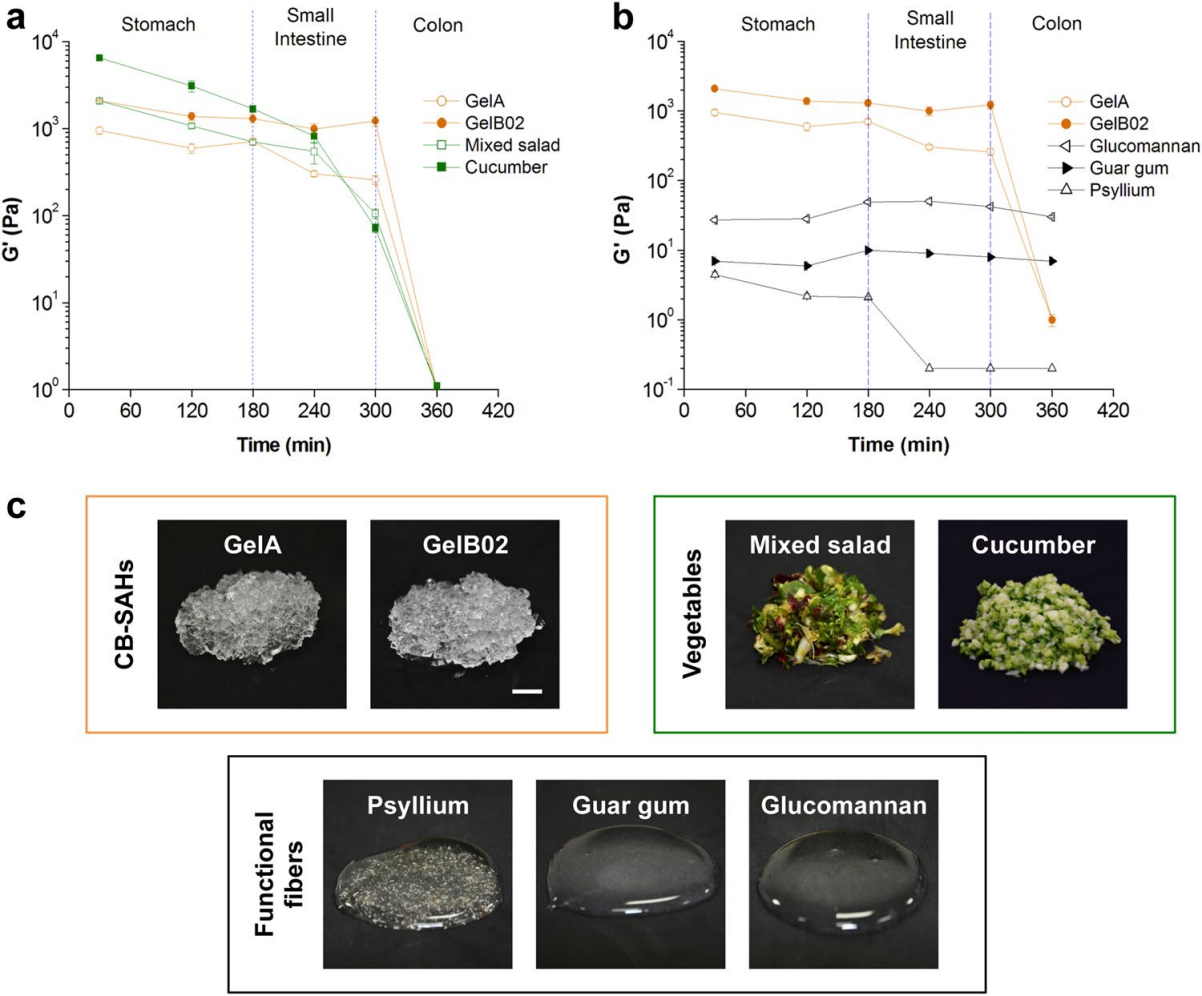
Comparing the mechanical properties of CB-SAHs, vegetables and functional fibers in simulated GI conditions. Elasticity (G′) of GelA and GelB02 compared with that of (**a**) minced raw vegetables and (**b**) functional fibers, as assessed by means of an in vitro simulated GI model (n = 3 at each time point, mean ± SD; plots produced with Origin 6.0, www.originlab.com). (**c**) Visual appearance of hydrated CB-SAHs, minced vegetables (with a particle size of about 2 mm, comparable to hydrated CB-SAHs granules) and functional fibers (at 1% w/v) right before testing (scale bar 1 cm).

After assessing the performance of GelA and GelB02 in simulated GI conditions, we used the same model to compare the elasticity of the CB-SAHs to two reference raw vegetables, minced cucumber and mixed salad, and three functional fibers, glucomannan, guar gum and psyllium, through the GI tract simulation (Fig. 5a, b). We monitored the G′ of the two CB-SAHs, as well as the reference vegetables and fibers as a function of the changing environmental GI conditions. The measured values of G′ variation generated distinctive profiles of behavior of the tested materials through time in the simulated conditions.

As reported in Fig. 5a, the elasticity profiles of the CB-SAHs and the raw vegetables were very similar. In detail, throughout the incubation in SGF the G′ of GelB02 (about 1300–2000 Pa) was maintained in the range defined by the modulus of mixed salad (as lower limit) and that of cucumber (as upper limit), while the G′ of GelA (about 600–950 Pa) was kept below the modulus of mixed salad. However, in the time frame between 180 and 240 min (i.e. between the final stage of SGF digestion and the initial stage of SIF digestion), both GelA and GelB02 showed very close similarity with the tested vegetables. More precisely, GelA showed G′ values very similar to those of mixed salad (at 180 min, about 705 Pa vs. 698 Pa for GelA and salad respectively, *p* = 0.95; at 240 min, about 302 Pa vs. 544 Pa for GelA and salad, *p* = 0.05). As regards GelB02, although at 180 min it showed an elastic modulus still lower than that of cucumber (about 1300 Pa vs. 1687 Pa for GelB02 and cucumber, *p* = 0.002), at 240 min the G′ moduli of GelB02 and cucumber were comparable (about 994 Pa vs. 816 Pa for GelB02 and cucumber, respectively; *p* = 0.15). It is also worth noting that, upon incubation in SIF, the G′ of both vegetables tended to decrease quite rapidly; at 300 min, cucumber and salad showed similar G′ values (~ 815 Pa for cucumber and ~ 544 Pa for mixed salad, *p* = 0.78). On the contrary, the elasticity of GelA and GelB02 appeared quite stable in SIF. However, upon incubation in SCF, both vegetables and CB-SAHs lost their elasticity, due to degradation.

In contrast, glucomannan, guar gum and psyllium, showed a completely different elasticity profile (Fig. 5b). All the tested functional fibers demonstrated orders of magnitude lower G′ modulus (about two orders of magnitude) than the CB-SAHs, in both SGF and SIF. Glucomannan, which was the fiber with the highest G′ among the tested functional fibers, showed G′ values in the range 27–50 Pa through the different media. The G′ modulus of guar gum showed a similar trend to glucomannan, although its values were kept in the lower range 6–10 Pa. Interestingly, in SCF, both glucomannan and guar gum were quite stable, compared to the CB-SAHs. With regard to psyllium, it showed very low values of G′, lower than 5 Pa, and was found to undergo a rapid degradation in SIF.

### Ex vivo organ culture

The potential impact of the CB-SAHs on the gut tissue was studied by utilizing an ex vivo organ culture (EVOC) system (as described in the Methods)^36^. Since weight management interventions are typically long term, understanding the effects of the CB-SAHs on the gut health could further teach us about the potential safety and efficacy of these products. In particular, we wanted to investigate whether the gut tissue would interact differently with hydrogels having similar composition but different elasticity levels, using as a reference the same vegetables and functional fibers studied in the GI simulation. For this purpose, we tested 4 types of CB-SAHs with variable elasticity, namely GelB01, GelB02, GelB03 and GelB04 (Table 1). While these CB-SAHs are all based on CMC-H, their elasticity progressively increases from GelB01 to GelB04, as highlighted in Fig. 3d, e.

**Figure 6:**
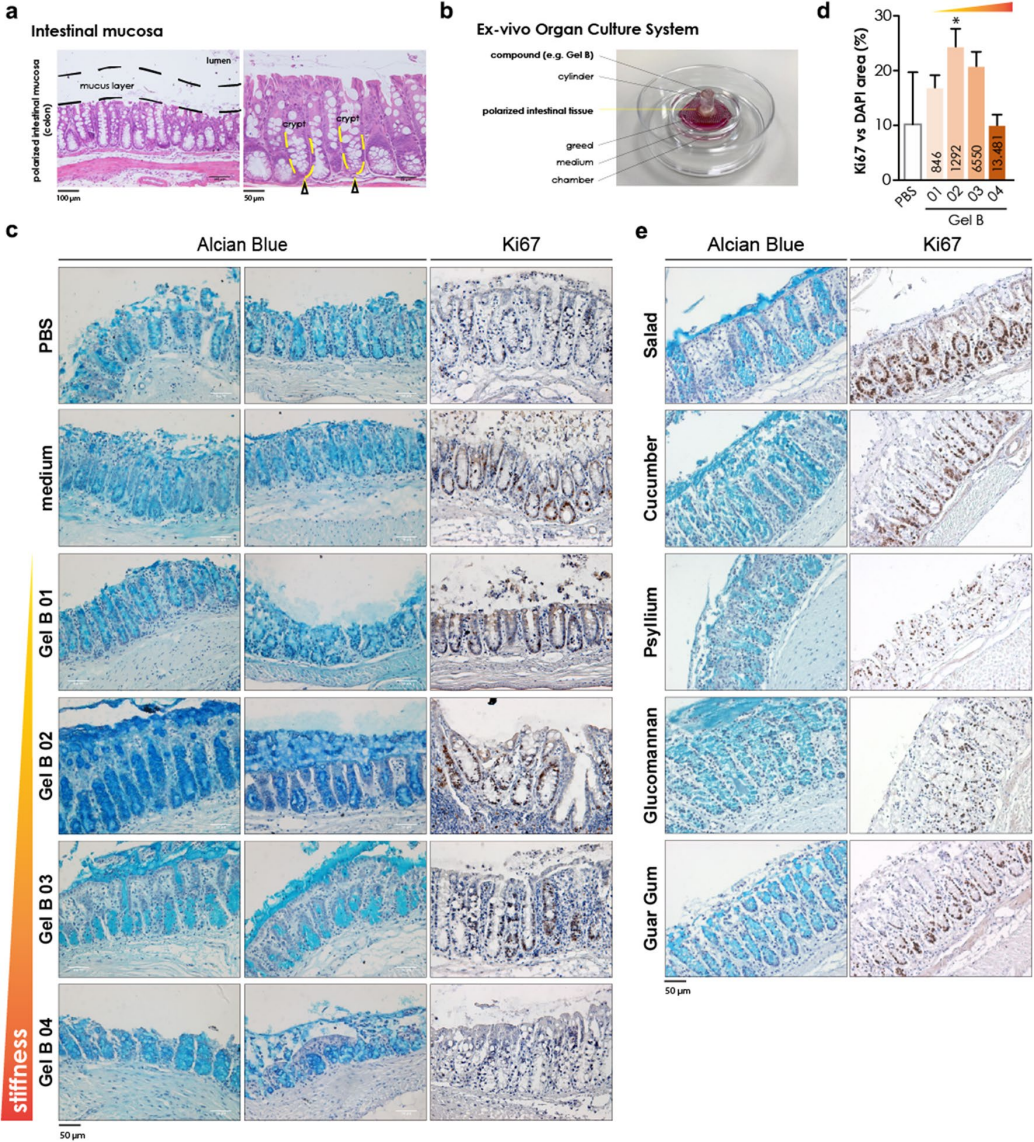
Results of the EVOC study. (**a**) Hematoxylin and eosin staining of colonic tissues. The mucus layer area (not stained) is indicated in black in the left panel; the right panel shows a magnification of the intestinal mucosa (strongly stained by the eosin, pink; nuclei are stained with hematoxylin, light blue): some crypts are highlighted in yellow, while the yellow arrows indicate the base of the crypts, containing the highest number of proliferating cells in the healthy tissue. (**b**) Ex vivo Organ Culture System. (**c**; **e**) Alcian Blue staining and Ki67 staining of colonic tissue EVOC specimens. The mucus is stained in blue; Ki67 positive (proliferating) nuclei are in brown; total nuclei in all the images are counterstained with hematoxylin. (**d**) Quantification of the Ki67 staining (expressed as Ki67-positive area over total nuclei area) reported in panel (**c**); values in the bars refer to the gel elasticity (G′), while *indicates significant difference (*p* < 0.05) with respect to the medium.

A schematic representation of the intestinal epithelium, the mucus layer and the proliferating cell niche is represented in Fig. 6a (yellow arrows), while Fig. 6b shows the EVOC system, with the compounds in direct contact with the explanted tissues. When testing the CB-SAHs (upon hydration in PBS), it emerged that the mucus layer as well as the tissue architecture were preserved, in a way comparable to the medium or even better (the blue staining represents the mucus layer, in Fig. 6c), when the tissue was in contact with GelB02 or GelB03, i.e. the CB-SAHs having an intermediate elasticity level, approximately between 1300 and 6500 Pa (as shown in Fig. 3e). Conversely, GelB01 (i.e. the CB-SAH with the lowest elasticity, about 800 Pa) and GelB04 (i.e. the CB-SAH with the highest elasticity, about 13,000 Pa) failed in maintaining the mucus integrity, similarly to PBS (Fig. 6c). In agreement with the effect on mucus layer, GelB02 and GelB03 performed better in preserving the tissue proliferative ability, similarly to the medium (brown nuclei correspond to proliferating cells, in Fig. 6c; total nuclei are counterstained with hematoxylin). On the contrary, proliferative cells became rare in tissues exposed to GelB01 or GelB04, similarly to PBS (Fig. 6c; the quantification of Ki67 staining is reported in Fig. 6d).

We then compared GelB02, the hydrogel behaving more similarly to the medium condition (positive control), to the functional fibers and vegetables in the same conditions. Only the tested vegetables (both cucumber and salad) and glucomannan, which is the soluble fiber having the highest elasticity in PBS among those tested (Supplementary Fig. 3), were able to preserve the mucus (Fig. 6e) and partly the tissue architecture, similarly to GelB02; conversely, both psyllium and guar gum failed in fully preserving tissue homeostasis ex vivo (Fig. 6e). Moreover, while cucumber and salad were able to maintain tissue proliferation as GelB02, none of the fibers were able to do so; indeed, when the tissue was exposed to soluble fibers, the tissue damage was such that the Ki67 staining was of bad quality and could not be used reliably.

## Discussion

Here, we describe a novel CB-SAH platform developed as an oral non-systemic approach for treating obesity, which was designed taking inspiration from the composition and mechanical properties of raw vegetables. We hypothesized that such biomimetic approach could enable new treatments with favorable efficacy and safety profile. Unlike different superabsorbent hydrogels that have been previously reported, the products of this technology are entirely based on GRAS building blocks used in foods.

We demonstrate that, by a proper selection of the CMC type and a proper control of the CA crosslinking, stable CMC networks can be created, which show both high absorption capacity and tunable elastic response (e.g. GelA and GelB02) (Fig. 3). In general, for a given polymer backbone, an inverse inter-dependency exists between the degree of hydration and the elasticity of the material, as both hydration and elasticity are related to the crosslink density of the polymer network^30^. Here, we show that the CB-SAHs hydration and elasticity are inversely related when using a fixed CMC type, as expected, while being tuned almost independently of each other when using different CMC types (e.g. CMC-L and CMC-H for GelA and GelB02, respectively).

Moreover, the so-obtained CB-SAHs are sensitive to ionic strength and pH variations, thus can be tuned to function in the wide range of conditions found in the GI tract. Utilizing an in vitro GI model (Fig. 4a, b), we identified the potential mechanism by which orally administered CB-SAHs, taken before meals, work to enhance satiety and assist in weight management (Fig. 4c):

- Gastric soluble capsules rapidly degrade in the stomach, releasing thousands of CB-SAHs granules.
- The granules hydrate up to about 100 times their original volume, with each hydrated granule becoming a small gel piece without caloric value, with elasticity level similar to ingested raw vegetables.
- The thousands of the gel pieces do not cluster, but mix homogeneously with the ingested foods and reduce the caloric density of the meal.
- After further digestion, when the pH is reduced due to the excretion of gastric fluids, the CB-SAHs granules shrink, thus are more easily cleared from the stomach together with the food.
- Once in the small intestine, the granules hydrate again in the higher pH environment, increasing both the volume and the elasticity of the bolus.
- When arriving to the colon, similarly to ingested vegetables, the gel pieces are partially degraded through enzymatic activity, thereby releasing the carried water which is re-absorbed by the body, while the cellulosic material is excreted with the feces.

Importantly, the CB-SAHs remain undigested in the stomach and the small intestine, therefore are not absorbed systemically. They work only through mechanobiological modes of action (by increasing the volume and elasticity of the ingested food) and by reducing the caloric density of meals. In this regard, our in vitro GI model also demonstrated that the tested CB-SAHs (GelA and GelB02) had elasticity levels, when in the stomach and small intestine, remarkably similar to some raw vegetables (cucumber and salad) in those environments (Fig. 5a). Therefore, in accordance with our biomimetic approach, CB-SAHs were able to emulate basic compositional and mechanical features of raw vegetables, as further described in Table 2^11,37–40^. Interestingly, the elasticity of CB-SAHs and vegetables starkly contrasted to some functional fibers (glucomannan, guar gum and psyllium), which demonstrated orders of magnitude lower elasticity throughout the GI simulation (Fig. 5b). This significant difference of elasticity is mainly due to the fact that CB-SAHs and vegetables carry and hold water inside their tridimensional structure, behaving as solids, while functional fibers carry water on the surface of their linear structure, behaving as liquid solutions (Fig. 5c).

**Table 2:** Comparing basic compositional and structural features of common vegetables (salad and cucumber) and CB-SAHs (GelA and GelB02).

	Cellulose type/solubility	% Cellulose* (%)	% Water* (%)	Gastric volume** (mL)	G′ range (kPa)	
					SGF	SIF
**Vegetables**						
Salad	Native Insoluble	0.9	96.0	~ 375	0.69–2.07	0.10–0.69
Cucumber	Native Insoluble	0.6	96.0	~ 375	1.68–6.49	0.07–1.68
**CB-SAHs**						
GelA	X-linked CMC-L Insoluble	1.1	98.9	~ 385	0.70–0.94	0.25–0.70
GelB02	X-linked CMC-H Insoluble	1.3	98.7	~ 350	1.30–2.08	1.22–1.30

The table compares values of cellulose and water content, gastric volume achieved by the recommended daily intake, and ranges of elastic modulus G′ as measured in vitro in SGF and SIF.

*Water and cellulose contents in vegetables are taken from the literature^11,37,38^.

**Volume of vegetables is estimated based on the daily recommended amount (375 g) by the American Heart Association^39^, while the volume of CB-SAHs is calculated based on the daily dose (4.5 g) of a CB-SAH product (Gelesis100) according to its use in clinical setting^40^.

In order to further understand the importance and relevance of the mechanical properties versus the composition of CB-SAHs, vegetables and fibers, we utilized the EVOC experimental model (Fig. 6). We found that intestinal mucosa homeostasis and tissue integrity preservation were optimal, and similar to those observed for the medium control, only when the tissue was exposed to CB-SAHs of specific range of elasticity levels (GelB02 and GelB03). Cucumber and salad, which have mechanical properties similar to GelB02, were also able to preserve both the mucus layer and the proliferative niche in the colonic mucosa, in contrast to functional fibers, which have much lower elasticity. These results suggest that it is not just the chemical composition, but also the mechanical properties of the GI content that play an important role in the maintenance of gut tissue health. This opens to a broader area of research on tissue-material mechanical interactions and the potential use of mechanotransduction based therapeutics in gut related diseases.

Importantly, the CB-SAHs are not intended to be used as a replacement for vegetables, as they do not contain any micronutrients. The intention behind this biomimetic approach was to create an orally administered, non-systemic tool for weight management, acting mainly through mechanobiological modes of action. At the time of this publication, clinical studies performed on a CB-SAHs product from this platform^40,41^ and resulting regulatory clearances, in the US and Europe, show that these hydrogels represent a useful aid in weight management for the treatment of individuals with overweight and obesity when used in conjunction with diet and exercise. Notably, there is no limit on treatment duration, likely due to the favorable safety profile of this approach. No serious adverse events (AEs) were observed in the CB-SAH group. The most common (> 5%) gastrointestinal AEs in the CB-SAH group were abdominal pain, constipation, flatulence, infrequent bowel movements, abdominal distension, diarrhea and nausea. Ninety-five percent of AEs were mild or moderate in intensity, occurred within the first 3 months and resolved within 2 weeks. Further indications, related to metabolic diseases and gut health, are being explored, utilizing several hydrogel candidates from this platform.

## Conclusions

A novel CB-SAH technology was developed taking inspiration from the composition and mechanical properties of ingested raw vegetables. This biomimetic approach enabled the development of a new validated therapeutic tool to aid in weight management, with a highly favorable efficacy and safety profile. Furthermore, results from an EVOC study indicated that the mechanical properties of the biomimetic CB-SAHs could play an important role in the maintenance of gut tissue health. While further investigation is needed to understand the effects of the CB-SAHs mechanical properties on the gut tissue and the underlying mechanisms of action, these findings suggest a potential use of the CB-SAHs to also treat other conditions affected by the gut health.

## Materials and methods

### Materials

Two types of pharmaceutical grade carboxymethylcellulose sodium salt (CMC) were utilized, referred to as CMC-L and CMC-H for lower and higher molecular weight (M_w_), respectively (Table 1; Supplementary Fig. 1). Both CMCs were provided by Eigenmann & Veronelli SpA and analyzed before further use (see Supplementary Methods). Pharmaceutical grade citric acid (CA) was purchased from Carlo Erba Srl. Functional fibers based on glucomannan 95% and psyllium were purchased from a local pharmacy (Lecce, Italy). Cucumber (*Cucumis sativus* L.) and mixed salad (*Lactuca sativa* L.) were provided by a local grocery store (Lecce, Italy). The use of cultivated plants in this study was in compliance with relevant institutional, national and international guidelines and legislation. All other materials and chemicals were supplied by Sigma Aldrich Srl, unless otherwise noted, and used as received.

### CB-SAHs synthesis

CB-SAHs were synthesized based on a proprietary technological platform, as previously described^42–44^. For the synthesis of GelA, a homogeneous mixture of CMC-L and CA in purified water (CA/CMC 0.3% w/w) was obtained through planetary mixing. The mixture was then dried, ground by a cutting mill, sieved and crosslinked at 120 °C for 8 h. The crosslinked powder was then washed in deionized water, filtered, dried, ground and sieved again, for a final particle size in the range 100–1000 μm.

The synthesis of GelB, based on CMC-H, was performed according to the same procedure described above, but using a lower CA/CMC weight ratio (0.002), in order to slow down the kinetics of the crosslinking reaction. Then, with the aim of producing GelB samples with different levels of elasticity, the crosslinking time was varied from 4 to 8 h. Dried, non-crosslinked material (GelB01) was also produced and used as a reference.

### Determination of medium uptake ratio (MUR)

The CB-SAHs hydration capability was preliminarily assessed in two different aqueous solutions, i.e. simulated gastric fluid (SGF) diluted with water (SGF/water 1/8 v/v, pH 2.1) and phosphate buffered saline (PBS, pH 7.4) (SGF was prepared according to USP specifications, as detailed in Supplementary Table 1). The first medium was selected to simulate the environmental conditions encountered by the CB-SAHs in the stomach, upon ingestion of a capsule with two cups of water. Measurements in SGF/water 1/8 v/v were performed at 25 °C. PBS was used as an inert control medium in the ex vivo experiments. In case of PBS, measurements were performed at 37 °C.

The CB-SAHs hydration was evaluated by incubating 0.25 g of dry granular CB-SAHs samples in 40 mL of the selected medium (1/160 w/v) under gentle stirring, for 30 min. The CB-SAHs powder was then collected, drained on a filter to eliminate excess water and weighed. The medium uptake ratio (MUR) was calculated as follows:

1 $${\text{MUR}} = \left( {{\text{W}}_{{\text{f}}} - {\text{W}}_{{\text{i}}} } \right){\text{/W}}_{{\text{i}}}$$

where W_f_ is the final weight of the hydrated gel and W_i_ is the initial weight of the dry gel. Three samples from independent batches were tested for each CB-SAHs type.

### Determination of elasticity via dynamic-mechanical analysis (DMA)

The elasticity of the hydrated CB-SAHs was evaluated right after the determination of the MUR, by means of dynamic-mechanical analysis (DMA). The hydrated CB-SAHs particles were placed between the parallel plates (cross-hatch configuration, diameter 40 mm) of a rotational rheometer (Discovery HR-1, TA Instruments). The gap between the plates was set to 4 mm. A preliminary strain sweep test was performed to select a strain value where linear viscoelasticity is observed. Then the material was subjected to a frequency sweep test in the range 1–50 rad/s at 0.1% strain. The value of the elastic or conservative modulus G′ at a frequency of 10 rad/s was used to compare the elasticity of the different types of CB-SAHs. Corresponding values of the viscous or dissipative modulus G” were also recorded to fully characterize the dynamic-mechanical behavior of the CB-SAHs. For each medium and CB-SAHs type, measurements were run in triplicate.

### Determination of MUR and elasticity in simulated GI conditions

In order to simulate the CB-SAHs performance through the different parts of the GI tract, an in vitro model was designed to evaluate the MUR and G′ in a reproducible system. Samples of GelA and GelB02 were sequentially incubated in different aqueous media at 37 °C (1/160 w/v), under mild mechanical stirring, for selected time lengths (roughly corresponding to the expected transit times in vivo) (Supplementary Table 2). The aqueous media are meant to mimic the various environments found in the GI upon digestion, in terms of local pH and enzyme content. Simulated gastric fluid (SGF, pH 1.1), simulated intestinal fluid (SIF, pH 6.8) and simulated colonic fluid (SCF, pH 6.8) were prepared according to USP specifications (Supplementary Table 1). SGF/water 1/8 v/v (pH 2.1) was used to simulate the initial stomach environment encountered by the CB-SAHs, up to 60 min of incubation. To simulate the later stages of stomach digestion, when the pH further lowers^45^, the CB-SAHs were then immersed in SGF/water 1/4 v/v (pH 1.8), up to 120 min, and in pure SGF, up to 180 min. The transit in the intestine was then simulated by incubating the materials in SIF, up to 300 min, followed by immersion in SCF, up to 360 min. Upon incubation in the different media, at given time points (5, 15, 30, 60, 120, 180, 240, 300, 360 min) the CB-SAHs samples were recovered by filtration and weighed to measure the MUR. Then, right after the MUR evaluation, samples were also tested via DMA, as described above, to estimate the elastic modulus G′. A total of 9 samples (one for each time point) were assessed for a given simulated GI test, and the test was repeated 3 times using CB-SAHs samples from three independent batches.

### Mechanical properties comparison with raw vegetables and functional fibers in simulated GI conditions

In order to compare CB-SAHs to vegetables and functional fibers in the simulated GI test, the following samples were prepared: 2 CB-SAHs samples, i.e. GelA and GelB02, 2 vegetables samples, i.e. cucumber and green salad, and 3 functional fibers samples, i.e. glucomannan, guar gum and psyllium. The vegetables were freshly minced with a chopper to simulate the mechanical action of mastication and to obtain small particles, with an average size of about 2 mm (roughly comparable to the size of hydrated CB-SAHs granules). Then, minced vegetables were hydrated (1/160 w/v) in SGF/water 1/8 v/v and then incubated at 37 °C in the different aqueous media used to simulate the GI environment (Supplementary Table 2). At fixed time points (30, 120, 180, 240, 300 and 360 min), remnants of samples were recovered by filtration and assessed via DMA analysis to evaluate their elastic modulus G′, as described above. The GI simulation was repeated three times.

The functional fibers, unlike CB-SAHs and vegetables, are mostly soluble in aqueous media and cannot be serially recovered and immersed in different solutions. The experimental procedure used to measure the modulus G′ as a function of the GI environment was changed accordingly. For testing G′ at given time points (30, 120, 180, 240, 300 and 360 min), fiber samples were directly hydrated and incubated at 37 °C in the corresponding GI medium (1% w/v), for the expected residence time. The concentration of 1% w/v was chosen to test approximately the same mass of polymer between the DMA plates, with respect to the CB-SAHs. The test was conducted three times.

### Ex vivo organ culture (EVOC)

In order to study the effect of CB-SAHs with different elasticity levels on the structure of the gut mucosa, as well as to compare them to the effect of vegetables and functional fibers, we performed an ex vivo organ culture of colon tissues explanted from 8 to 12 weeks old healthy C57BL6/J male mice (n = 6) (Charles River Laboratories), as previously described^36^. Mice were housed under specific-pathogen-free (SPF) conditions at Campus IFOM-IEO (Milan, Italy) and Humanitas Clinical and Research Center (Rozzano, Milan, Italy). All animal experiments were performed under protocols (n.31/13, 139/15, 532/17) approved by the Italian Ministry of Health, and consistent with national (D.L. N. 26, G.U. March 4, 2014) and international law and policies (EEC Council Directive 2010/63/EU), in accordance with ARRIVE guidelines.

The clean mucosa was cut into 1 cm^2^ pieces and a cave cylinder was glued on the apical face of the mucosa. The mucosa was then placed on a sterile metal grid in a center well organ culture dish filled with a nutrient rich culture media (DMEM, 15%FBS, glutamine, epidermal growth factor and Insulin-Transferrin-Selenium-X). Upon mucus reconstitution (1 h at 37 °C), colon tissues were incubated for 2 h at 37 °C with CB-SAHs with variable elasticity (Table 1, Fig. 3e), and with functional fibers (i.e. glucomannan, guar gum and psyllium) dissolved in PBS (2% w/v) filling the cave cylinder. Similarly, colon tissue samples were incubated with fresh cucumber and salad previously chopped and partially smashed and placed in the cylinder with a spatula*.* PBS and medium treated tissues were used as controls.

At the end of incubation samples were fixed and embedded and sectioned for following analysis.

Sections were hence stained with Alcian Blue/PAS (to visualize the mucus and mucus-secreting cells) or with Ki67 antibody (to detect cell proliferation), as briefly described in the following.

### Carnoy’s and PFA fixation

To preserve mucus layer, half of the tissue was fixed in Carnoy’s fixative (Ethanol, Acetic Acid Glacial, Chloroform 6:1:3). After 2 h of fixation, tissues were transferred in absolute ethanol, processed and paraffin embedded. To preserve the tissue structure to perform histological analysis, half of the tissue was fixed in PFA 4% (Paraformaldehyde). After overnight fixation, tissues were transferred in ethanol 70%, processed and paraffin embedded.

### Alcian Blue mucus staining

Tissues were then stained using Alcian Blue-PAS ready to use staining kit (NovaUltra™ Alcian Blue/PAS Stain Kit, IHC WORLD) following provider’s instructions; Alcian blue will stain strongly acidic mucins in blue.

### Immunohistochemistry for Ki67

Immunohistochemistry for Ki67 was performed on 4% PFA or Carnoy fixed paraffin-embedded tissues. Tissue sections were deparaffinized in xylene and hydrated through graded alcohol series. Antigen unmasking was performed using Tris-EDTA pH 9 at 95 °C for 50 min (PFA) or Citrate Buffer pH 6 (Carnoy) at 95 °C for 20 min, followed by quenching of endogenous peroxidases using 3% H_2_O_2**.**_ Sections were then incubated with primary rabbit polyclonal antibody against Ki67 (ab15580, ABCAM) for 1 h at room temperature and with secondary antibody ready to use (DAKO Envision system HRP rabbit) for 20 min at room temperature. Tissue sections were then washed and incubated with peroxidase (DAB, DAKO) solution. Slides were then counterstained with hematoxylin. Images were acquired using Olympus BX51 Widefield microscope connected to a Nikon DS-5M camera. Ki67 staining was quantified by using ImageJ software and Immunoratio plugin^46^.

### Statistical analysis

All data were expressed as mean ± standard deviation (SD), unless otherwise noted. Statistical significance was determined by using ANOVA and Fisher’s PLSD tests, and differences were considered to be significant when *p* value < 0.05.

## Supplementary Information

Supplementary Video 1.

Supplementary Information.

## Data Availability

Further information on the datasets generated and/or analyzed in this work is available from the authors upon reasonable request.
