# Author Correction: Biomimetic cellulose-based superabsorbent hydrogels for treating obesity

**DOI:** 10.1038/s41598-021-04527-7

**Published:** 2021-12-29

**Authors:** Marta Madaghiele, Christian Demitri, Ivo Surano, Alessandra Silvestri, Milena Vitale, Eliana Panteca, Yishai Zohar, Maria Rescigno, Alessandro Sannino

**Affiliations:** 1 Department of Engineering for Innovation, University of Salento, Via per Monteroni, 73100 Lecce, Italy; 2 IRCCS Humanitas Research Hospital, Via Manzoni 56, 20089 Rozzano, Milan, Italy; 3 Gelesis, Boston, MA 02116 USA; 4 Gelesis, 73021 Calimera, Lecce Italy; 5 Department of Biomedical Sciences, Humanitas University, Via Rita Levi Montalcini 4, 20072 Pieve Emanuele, Milan Italy

Correction to: *Scientific Reports* 10.1038/s41598-021-00884-5, published online 01 November 2021

The original version of this Article contained an error in Affiliation 2, which was incorrectly given as ‘Mucosal Immunology and Microbiota Unit, Humanitas Research Hospital, 20090, Pieve Emanuele, Milan, Italy’. The correct affiliation is listed below:

IRCCS Humanitas Research Hospital, Via Manzoni 56, 20089, Rozzano, Milan, Italy

In addition, an affiliation for Maria Rescigno was omitted. The correct affiliations are listed below.

IRCCS Humanitas Research Hospital, Via Manzoni 56, 20089, Rozzano, Milan, Italy

Department of Biomedical Sciences, Humanitas University, Via Rita Levi Montalcini 4, 20072 Pieve Emanuele, Milan, Italy

The original Article and accompanying Supplementary Information file have been corrected.

