# Supplementary material for: Biomimetic cellulose-based superabsorbent hydrogels for treating obesity
Source: Sci Rep. 2021 Nov 1;11:21394. doi: 10.1038/s41598-021-00884-5 (PMC8560814; doi:10.1038/s41598-021-00884-5)
Supplement: Supplementary file 2 — Supplementary Information. [file 41598_2021_884_MOESM2_ESM.pdf]

Supplementary Information

BIOMIMETIC CELLULOSE-BASED SUPERABSORBENT HYDROGELS FOR TREATING OBESITY

Marta Madaghiele[1*], Christian Demitri[1], Ivo Surano[1], Alessandra Silvestri[2], Milena Vitale[2], Eliana Panteca[3,4], Yishai Zohar[3,4], Maria Rescigno[2,5], Alessandro Sannino[1*]

[1] Department of Engineering for Innovation, University of Salento
Via per Monteroni, 73100 Lecce, Italy

[2] IRCCS Humanitas Research Hospital
Via Manzoni 56, 20089 Rozzano, Milan, Italy

[3,4] Gelesis
Boston, MA, 02116 USA & Calimera, Lecce 73021, Italy

[5] Department of Biomedical Sciences, Humanitas University
Via Rita Levi Montalcini 4, 20072 Pieve Emanuele, Milan, Italy

[*]Corresponding authors:
Marta Madaghiele
Department of Engineering for Innovation, University of Salento
Via per Monteroni, Lecce 73100, Italy
Email: marta.madaghiele@unisalento.it

Alessandro Sannino
Department of Engineering for Innovation, University of Salento
Via per Monteroni, Lecce 73100, Italy
Email: alessandro.sannino@unisalento.it

1. Supplementary Methods

Analysis of CMC types

Size exclusion chromatography (SEC)

SEC was performed to assess the molecular weight distributions of CMC-L and CMC-H. A Waters e2695 liquid chromatograph coupled with Waters 2414 differential refractometer detector (RID) were used for the measurements. Separation was performed in two Agilent PL-aquagel-OH Mixed-H columns (300 x 7.5 mm, 8 µm particle size) in series, preceded by Agilent PL-aquagel-OH Guard column (50 x 7.5 mm, 8 µm particle size). A pH 7 aqueous buffer (0.2 M sodium nitrate and 0.01 M sodium phosphate monobasic dihydrate in purified water) was used as the mobile phase at a flow rate of 1 mL/min and 35°C. A calibration curve was obtained by analyzing a series of pullulan standards (Ready Cal-Kit Pullulan high, PSS Polymer Standards Service GmbH) in the molecular weight range from 6,100 to 1,220,000 g/mol, solubilized in purified water at a concentration of 0.15% w/w. CMC samples (n=4 for each type), dissolved in purified water overnight at a concentration of 0.15% w/w, were then analyzed. Data were collected and processed using the Empower3 software (Waters Corporation) to determine the weight average molecular weight (M_w), the number average molecular weight (M_n) and the polydispersity index ($PDI=M_w/M_n$), by means of the integration algorithm ApexTrack.

Rheological measurements (hysteresis loop tests)

For each CMC type, rheological measurements on CMC solutions (2% w/v) were performed at 25°C, using a Discovery HR-1 rotational rheometer (TA Instruments) with the cone-and-plate geometry (diameter 40 mm, gap 0.026 mm). In order to evaluate the thixotropic behavior of the CMC solutions, a hysteresis loop test was carried out, which consisted of the following loading/unloading program, under controlled shear rate mode: (a) increase of the shear rate from 0.1 to 100 s^{-1} in 5 minutes; (b) constant shear rate of 100 s^{-1} for 1 minute; (c) decrease of the shear rate from 100 to 0.1 s^{-1} in 5 minutes. Considering the plot of shear stress *vs.* shear rate, the upward (loading) and the downward (unloading) curves do not superimpose, with the downward curve being located below the upward one. A dimensionless thixotropic index (TI) was thus calculated as:

$$TI = \frac{A_{up} - A_{down}}{A_{up}} \times 100 \qquad \text{(Eq. S1)}$$

where A_{up} and A_{down} are the areas under the upward and the downward curves, respectively, and (A_{up} - A_{down}) represents the hysteresis area.

2. Supplementary Figures (Supplementary Figs. 1-3)

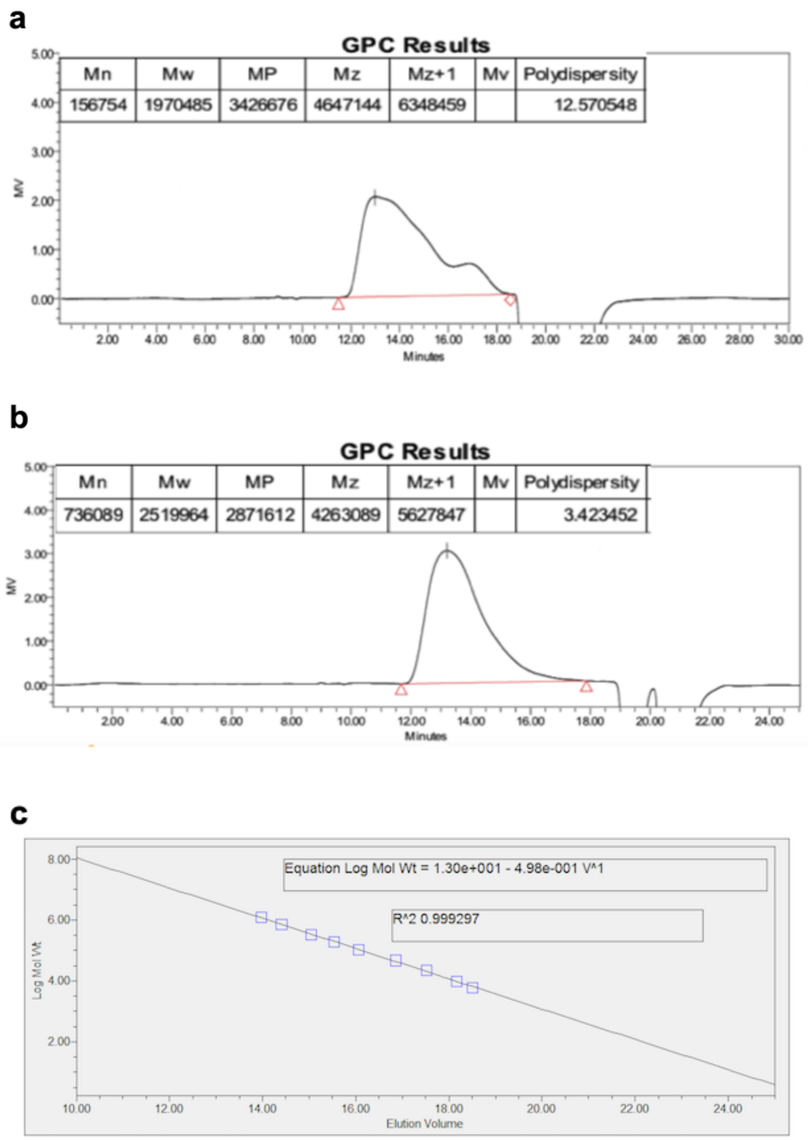

Supplementary Fig. 1. Results of size exclusion chromatography.

Typical chromatograms of CMC-L **(a)** and CMC-H **(b)**, indicating a larger M_w and a narrower molecular weight distribution of CMC-H compared to CMC-L. **(c)** Calibration curve obtained from pullulan standards and used for the analysis of the CMC samples.

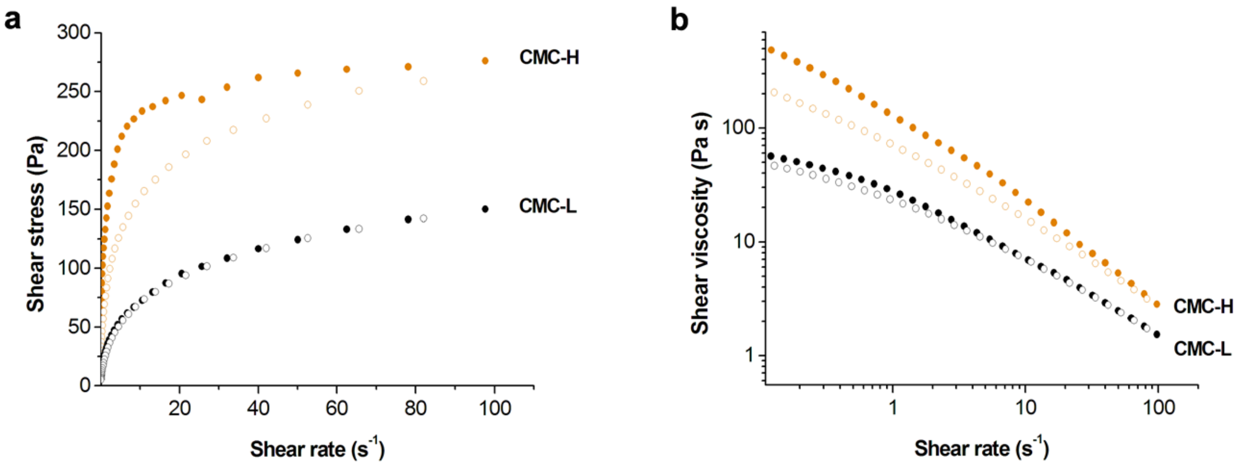

Supplementary Fig. 2. Results of hysteresis loop tests performed on aqueous CMC solutions (2% w/v).

Typical plots of shear stress **(a)** and shear viscosity **(b)** of CMC-L and CMC-H solutions, as a function of the shear rate: full symbols indicate the upward/loading curve, while empty symbols indicate the downward/unloading curve. The hysteresis area in **(a)** gives an idea of the extent of thixotropy, which is calculated according to Eq. S1.

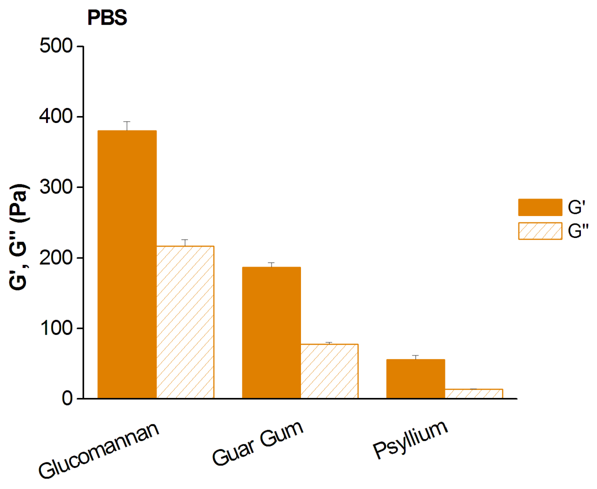

Supplementary Fig. 3. Elastic modulus G' and viscous modulus G'' (at 10 rad/s) of functional fibers upon solubilization in phosphate buffered saline (PBS) at 37°C (2% w/v).

Results are the mean ± SD of three independent measurements. All detected differences in elasticity (G') among glucomannan, guar gum and psyllium were significant (p<0.0001).

3. Supplementary Tables 1-2

Supplementary Table 1. Simulated Gastric Fluid (SGF), Simulated Intestinal Fluid (SIF) and Simulated Colonic Fluid (SCF) used in this study.

* Used enzymes were pepsin (1:3000), pancreatin from porcine pancreas (8xUSP) and pectinase from *Aspergillus niger* (>1 U/mg).

	SGF	SIF	SCF
**COMPOSITION**			
Sodium chloride (g)	2.0	-	-
Hydrochloric acid 1 M (mL)	84.0	-	-
Purified water (mL)	-	750.0	750.0
Monobasic potassium phosphate (g)	-	6.8	6.8
Sodium hydroxide 0.2 N (g)	-	117.0	117.0
Pepsin* (g)	3.2	-	-
Pancreatin* (g)	-	10.0	-
Pectinase* (g)	-	-	10.0
Adjust pH and/or volume			
Sodium hydroxide 0.2 N to (or hydrochloric acid 0.2N)	-	pH 6.8	pH 6.8
Purified water to (L)	1.0	1.0	1.0
**PROPERTIES**			
pH	1.1	6.8	6.8
Ionic strength (mM)	118	72	72

Supplementary Table 2. Simulated GI model to assess absorption (MUR) and elasticity (G') variations of the CB-SAHs at 37°C.

* Each time point represents an individual sample (e.g. the sample tested at 120 min is previously immersed in SGF/water 1/8 up to 60 minutes and then immersed in SGF/water 1/4 for additional 60 minutes, before MUR and G' assessment).

Simulated GI environment	Simulated GI interval (min)	Simulating GI medium	Testing time (min)*
Stomach	0-60	SGF/water 1/8	5
			15
			30
			60
	60-120	SGF/water 1/4	120
	120-180	SGF	180
Small Intestine	180-300	SIF	240
			300
Large Intestine	300-360	SCF	360

4. Supplementary Video

Supplementary Video 1. Time-lapse video of the hydration of a single CB-SAH granule (GelA in SGF/water 1/8 v/v) over 10 minutes.
